# Comparison of Sensor-Based and Audible Detection of Milking Liner Slips during Machine Milking of Dairy Cows

**DOI:** 10.3390/s24051361

**Published:** 2024-02-20

**Authors:** Matthias Wieland, Madeleine Eve Spellman, Kerry Lynn Case, Christina Marie Geary, Anja Sipka

**Affiliations:** Department of Population Medicine and Diagnostic Sciences, College of Veterinary Medicine, Cornell University, Ithaca, NY 14853, USA

**Keywords:** agreement, bovine, machine milking, teat

## Abstract

On-farm milk flow meter technology facilitates real-time assessment of individual cow milking observations and could be used to detect milking liner slips during machine milking of dairy cows. Here, we compared the accuracy of on-farm milk flow meters for detecting milking liner slips with that of audible detection and that of a portable vacuum recording system. Compared to audible detection methods, the on-farm milk flow meter facilitated the detection of milking liner slips with moderate accuracy. Using the vacuum recording system as the gold standard, the milk flow meter system failed to detect most of the liner slips, leading to poor agreement between the two devices. We conclude that the on-farm milk flow meter system tested here compared well with audible detection; however, when vacuum recordings were considered, we found significant levels of under-detection. Taken together, dairy operators may use the on-farm milk flow meter system to inform adjustments of the milking machine settings and monitor milking routine performance. However, the system is not suitable for monitoring short-duration vacuum fluctuations. Future research is warranted to optimize the sensor-based detection of milking liner slips.

## 1. Introduction

Mastitis is one of the most frequent diseases in dairy cattle, and this disease has detrimental effects on animal well-being and dairy industry profitability [1]. Most mastitis cases are caused by intramammary infections, which result from pathogenic bacteria entering the mammary gland through the teat canal. Researchers have long recognized the importance of the milking machine in the development of intramammary infection in dairy cattle [2,3,4]. Among the many machine milking factors that influence the risk of intramammary infection, milking machine liner slips are reportedly “the single most important factor” [5]. A milking machine liner slip is defined as “rapid air leakage past the mouthpiece of the milking machine liner”, occurring between the liner and the skin of bovine teats during machine milking [6]. Historically, milking machine liner slips have been detected by installing vacuum transducers to measure vacuum fluctuations [6,7,8] or by listening for audible signs such as the squawk of escaping air [9]. However, to our knowledge, vacuum transducers have not been employed to detect machine milking liner slips on a routine basis. Furthermore, audible detection of milking machine liner slips is laborious and time-consuming, making this assessment a costly process. This hampers our ability to routinely monitor milking machine liner slips in modern dairy operations. Recent advances in electronic on-farm milk meter technology, such as near-infrared technology, allow for the documentation of liner slips during individual cow milking observations. This innovation offers a unique opportunity to objectively monitor liner slips in real-time during individual cow milking observations. However, this ability has not been rigorously examined. Our study builds on existing applications of on-farm milk meter technology and addresses the paucity of research regarding its suitability to monitor milking machine liner slips. The objectives of this study were to assess the accuracy of on-farm milk meter technology for detecting milking machine liner slips and compare it with (1) audible detection and (2) a portable vacuum recording system. We hypothesized that electronic on-farm milk flow meters would identify milking liner slips with comparable accuracy to that of the portable vacuum recording system and to that of audible detection. Specifically, we set out to conduct two separate trials: trial 1 to compare the on-farm milk meter technology with audible detection and trial 2 to assess the accuracy of the on-farm milk meter technology and compare it with a portable vacuum recording system.

## 2. Materials and Methods

### 2.1. Trial 1

#### 2.1.1. Study Site

Trial 1 was carried out on 9 August 2022 at a commercial dairy farm located near Ithaca, New York. At the time of this study, approximately 4200 lactating Holstein cows were housed in free-stall pens, bedded with manure solids, and fed a total mixed ration. Herd and cow data (i.e., lactation number and stage of lactation (days in milk; DIM)) were compiled in a dairy management software program (version 24.1.949, Dairy Comp 305, Valley Agricultural Software, Tulare, CA, USA). Cows were milked 3 times daily at 1:00, 9:00, and 17:00 h in a 100-stall parallel rotary parlor (RP3100HD, DeLaval International AB, Tumba, Sweden). The parlor was equipped with flow-responsive vacuum regulation (DeLaval International AB, Tumba, Sweden). The flow-responsive vacuum regulation operated with a milkline vacuum of 42 kPa with a cluster milk flow rate of <2.3 kg/min and a milkline vacuum of 46 kPa with a cluster milk flow rate of >2.3 kg/min. The milking unit consisted of the cluster MC70 (DeLaval International AB, Tumba, Sweden) and a milking liner with a round barrel shape (LS-01 NC, DeLaval International AB). The pulsators (EP100, DeLaval International AB, Tumba, Sweden) were set to a pulsation rate of 60 cycles/min, a ratio of 65:35, and a side-to-side alternating pulsation. Automatic cluster removers were set as follows: a cluster remover milk flow threshold of 1.6 kg/min, a delay of 1 s, and a vacuum decay time of 1.5 s. The milk sweep was initiated 5.0 s after unit retraction and lasted for 2 s. Milking system settings and milking characteristics were monitored with a dairy farm management software program (DelPro Farm Manager, version 5.1, DeLaval International AB, Tumba, Sweden). The rotational speed of the milking parlor was 4.9 s/stall (490 s/complete rotation). The parlor was operated in two 12-h work shifts, each containing four milking technicians assigned to perform the following tasks at four different stations:

1. Cleaning all teats in lactating quarters with an automated teat brush (stall 1);
2. Manually forestripping 2 teats and applying teat disinfectant to all teats (stall 3);
3. Wiping all teats with an individual clean cloth towel (stall 14);
4. Attaching and aligning the milking unit (stall 20 for early- and mid-lactation cows, stall 25 for late-lactation cows).

This setup resulted in a dip contact time of 54 s, tactile stimulation (calculated as the sum of cleaning with the teat brush at station 1 and manual forestripping at station 2) of approximately 6 s, and a preparation lag time (i.e., time from the first tactile stimulation to the milking unit attachment) of approximately 93 s (early- and mid-lactation cows) or 118 s (late-lactation animals). The different premilking stimulation regimens for cows in different stages of lactation were implemented by the dairy farm’s herd manager in accordance with previous research showing that late-lactation cows benefit most from a longer preparation lag time [10]. The forced retraction of the milking unit was initiated at stall 90, resulting in a maximum milking unit-on time of 333 s (early- and mid-lactation animals) or 309 s (late-lactation cows).

#### 2.1.2. Data Collection

Data were obtained during one 9:00 a.m. milking session. For audible detection of machine milking liner slips, 3 observers were positioned at different stations of the rotary parlor and individually recorded the presence or absence of a milking liner slip. The observation areas for the 3 observers were as follows: observer 1 at the beginning of milking (the first 12 stalls following milking unit attachment), observer 2 in the middle of the milking (stalls 35 or 40 to stall 78), and observer 3 at the end of milking (stalls 79 to 90). A machine milking liner slip was considered present if an audible sign (i.e., squawk) of air leakage past the mouthpiece of the milking liner was detected for any duration. Regardless of whether observers detected a liner slip, the milking units were not adjusted to avoid interfering with sensor-based detection and facilitate data comparison with the normal milking operation. The observers were blinded to the results of the sensor-based detection. Prior to the study, the 3 observers were trained in the detection of an audible milking liner slip. Discrepancies in the assessment of an audible milking liner slip (i.e., duration of squawk and sound intensity) were discussed, and a consensus was reached.

**Figure 1:**
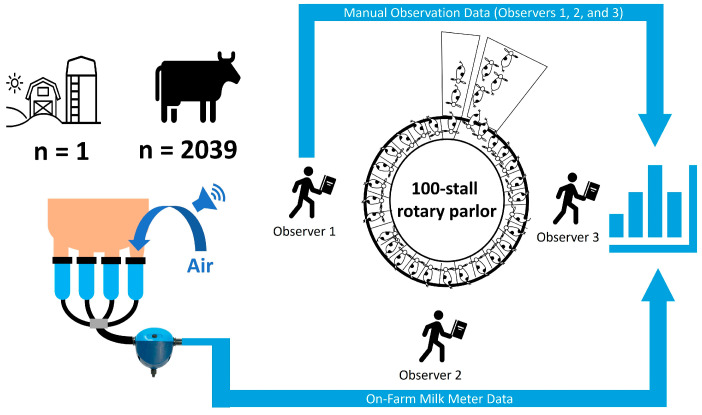
Study setup of trial 1. The presence or absence of audible liner slips in 2039 cow milking observations from a single dairy farm was assessed by 3 observers positioned at the beginning (observer 1), middle (observer 2), and end of milking (observer 3), together with automated detection through electronic on-farm milk flow meters. Subsequently, agreement and test statistics were calculated.

Data were collected on the following milking characteristics: total milk yield (yield of milk harvested from the start of milking to detachment of the milking unit, kg), 2-minute milk yield (amount of milk harvested within the first two minutes of milking, kg), milking unit-on time (duration from the start of milking to detachment of the milking unit, s), and average milk flow rate (total milk yield/milking unit-on time, kg/min). These data, along with the presence or absence of a machine milking liner slip, were obtained for each milking observation via electronic on-farm flow-through milk flow meters using near-infrared technology (MM27BC, DeLaval International AB, Tumba, Sweden) and recorded with the dairy farm management software (version 10, DelPro, DeLaval International AB, Tumba, Sweden). A report was created in DelPro that automatically recorded milk flow characteristics and exported them to a comma-separated value (csv) file. The operating principle of the MM27BC for the detection of a milking liner slip (SLP_MM_) is based on the amount of air in the milk. The sensitivity of the detection can be adjusted by means of a nondimensional threshold value, referred to as the ‘slip limit’, that ranges from 0 to 255, with the value 0 providing the highest sensitivity. The default threshold value is 140. A liner slip is recorded if the measured air bleed value remains above the threshold value for a minimum of 30 s. The recorded value is a binary value, whereas the time of occurrence, the duration of the milking liner slip, or the number of liner slips that occur during a single milking observation are not recorded. The slip limit was set to 175. An SLP_MM_ was therefore defined as present if an air leakage with an intensity above the threshold value of 175 was registered for a minimum of 30 s. Figure 1 illustrates the study design of trial 1.

### 2.2. Trial 2

#### 2.2.1. Study Site

Trial 2 was conducted from 1 March to 13 September 2023 at the Teaching Dairy Barn of the College of Veterinary Medicine, Cornell University (Ithaca, NY), and at the same commercial dairy farm as trial 1. The lactating herd of the Teaching Dairy Barn consisted of approximately 160 Holstein cows. Cows were also housed in free-stall pens with sand bedding and fed a total mixed ration. Herd and cow data (i.e., lactation number and stage of lactation (DIM)) were retrieved from Dairy Comp 305 (Valley Agricultural Software, Tulare, CA, USA). At the Teaching Dairy Barn, cows were milked 3 times per day at 4:00, 11:00, and 19:00 h in a double 10 parallel milking parlor (P2100, DeLaval International AB, Tumba, Sweden). The vacuum pump was set to supply a receiver operator vacuum of 45 kPa. The milking unit was composed of the cluster MC70 (DeLaval International AB, Tumba, Sweden) and a milking liner with a square barrel shape (ProSquare DPX2, IBA, Millbury, MA, USA). The pulsators (EP100, DeLaval International AB, Tumba, Sweden) were set to a pulsation rate of 60 cycles/min, a ratio of 70:30, and a side-to-side alternating pulsation. The automatic cluster removers were set to remove the units at a milk flow of 1.4 kg/min with a 0-s delay and a vacuum decay time of 2.3 s. The milk sweep was initiated 1.5 s after unit retraction and lasted for 4 s. The milking routine was performed by 2 operators per milking session. Premilking udder preparation was performed with sets of 5 cows in 5 steps and performed by milking operators in 3 visits.

1. Visit 1: step 1, wiping off teats with a clean, dry towel; step 2, applying teat disinfectant to all teats.
2. Visit 2: step 3, sequential forestripping of 3 streams of milk per quarter; step 4, wiping (i.e., dry and clean) of teats.
3. Visit 3: step 5, attachment of the milking unit.

This setup resulted in a tactile stimulation time (calculated as the sum of durations of forestripping and wiping) of approximately 9 s and a preparation lag time of approximately 70 s.

At the commercial dairy farm, the milking routine and machine settings were identical to those of trial 1, with one exception as follows: the flow-responsive vacuum regulation operated with a milkline vacuum of 38.0 kPa with a cluster milk flow rate of <1.6 kg/min and a milkline vacuum of 48.5 kPa with a cluster milk flow rate of ≥1.6 kg/min.

#### 2.2.2. Data Collection

**Figure 2:**
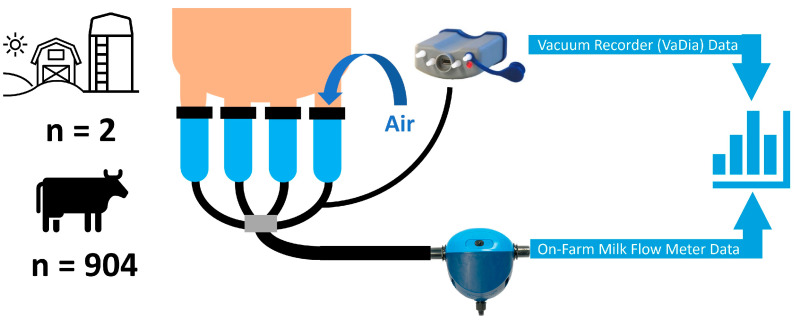
Study setup for trial 2. The presence or absence of milking liner slips in 904 cow milking observations from 2 dairy farms was assessed concomitantly with a portable vacuum recording device and electronic on-farm milk flow meters. Agreement and test statistics were calculated.

Data for trial 2 were collected during 15 visits (Teaching Dairy Barn, 11 visits during the 11:00 a.m. milking session; commercial dairy, 4 visits during the 9:00 a.m. milking session). Individual cow vacuum events were collected during milking using VaDia (BioControl, Rakkestad, Norway) vacuum recorders as previously described [11]. For this purpose, 4 VaDia devices were installed at the first 2 stalls on each parlor side at the Teaching Dairy Barn, and 10 VaDia devices were installed at 10 consecutive stalls at the commercial dairy farm. Vacuum recordings were reviewed and analyzed with the VaDia Suite software program (VaDia Suite, version 1.15.0.932; BioControl, Rakkestad, Norway) by 3 trained investigators who were blinded to the results of the milk flow meter recordings. Key events such as the start of milking, start of peak flow period, start of cyclic vacuum fluctuations, start of overmilking, start of take-off, and end of milking were determined through visual assessment rather than the automated detection of the software program. Subsequently, the following parameters were calculated with the software program: average cyclic vacuum fluctuations, overall average of average short milk tube vacuum calculations from ten pulsation cycles 60 s after the start of the peak flow period; irregular vacuum fluctuations type 1, a rapid drop of 100 kPa/second, and a magnitude of 21 kPa in short milk tube vacuum; and irregular vacuum fluctuations type 2, a rapid drop of 56 kPa/second and a magnitude of 14 kPa in short milk tube vacuum. In addition, data on the same milking characteristics as in trial 1, along with the presence or absence of a machine milking liner slip, were collected concomitantly via the electronic on-farm flow-through milk flow meters (MM27BC, DeLaval International AB, Tumba, Sweden) using the same settings as in trial 1. Figure 2 illustrates the study design of trial 2.

Prior to the study, the necessary sample sizes for both trials were calculated using Cohen’s κ statistic with the ‘irr’ package [12] in R (R Core Team [13]). The calculation was based on the frequency of milking liner slips detected audibly and with on-farm milk meters, specifically, the probability that the observers or the on-farm milk flow meter would detect a milking liner slip. We used data obtained with the dairy farm management software from previous milking sessions and assumed probabilities of 5% for both detection methods. A true Cohen’s κ statistic of 0.6, a value of κ under the null hypothesis of 0.4, a power of 0.8, an alpha-level of 0.05, and a two-sided test were employed. This calculation yielded a minimum sample size of 771 milking observations.

### 2.3. Data Analyses

Data from both trials were compiled in Microsoft Excel (2019 version, Microsoft Corp., Redmond, WA, USA) and JMP (version 15, SAS Institute Inc., Cary, NC, USA). Statistical analyses were performed in JMP and R. For the analyses of trial 1, four new binary variables (i.e., presence or absence of a milking liner slip) for the audible detection of a machine milking liner slip using the data from the 3 observers were created:

1. A liner slip detected by either observer 1 or observer 2 (SLP_OB1+2_);
2. A liner slip detected by either observer 1 or observer 3 (SLP_OB1+3_);
3. A liner slip detected by either observer 2 or observer 3 (SLP_OB2+3_);
4. A liner slip detected by any of the 3 observers (SLP_ANY_).

This stratified approach was used to describe possible temporal differences in the detection of liner slips and identify possible shortcomings of the sensor-based detection method. For the analyses of trial 2, two new binary variables (i.e., presence or absence of a milking liner slip) for the detection of a machine milking liner slip using the data from the VaDia device were created:

1. A liner slip detected by means of irregular vacuum fluctuation type 1 was defined as present if 1 or more events of irregular vacuum fluctuation type 1 were documented (SLP_V1_);
2. A liner slip detected by means of the irregular vacuum fluctuation type 2 was defined as present if 1 or more events of irregular vacuum fluctuation type 2 were documented (SLP_V2_).

To test the hypothesis that the electronic on-farm milk flow meter could identify milking liner slips, as verified through audible detection (i.e., trial 1), two different approaches were used. First, κ statistics were calculated to determine the individual agreement beyond that of chance of the binary variable SLP_MM_ with the binary variables SLP_OB1_, SLP_OB2_, SLP_OB3_, SLP_OB1+2_, SLP_OB1+3_, SLP_OB2+3_, and SLP_ANY_ using the package ‘DescTools’ in R [14]. The κ values were interpreted according to Landis and Koch [15] as follows: <0.21 poor agreement, 0.21–0.40 fair agreement, 0.41–0.60 moderate agreement, 0.61–0.80 good agreement, and 0.81–1.00very good agreement. Second, sensitivity, specificity, and positive (PPV) and negative predictive values (NPV) were calculated to evaluate the diagnostic performance of SLP_MM_ in detecting a milking liner slip as verified through manual assessment, using SLP_OB1_, SLP_OB2_, SLP_OB3_, SLP_OB1+2_, SLP_OB1+3_, SLP_OB2+3_, and SLP_ANY_ as the gold standards. The 95% confidence intervals (95% CI) for the sensitivity, specificity, and PPV and NPV were calculated as ± 1.96 × standard error, where standard error = √(a(1-a)/n), a is the test proportion, and n is the sample size. We interpreted test statistics ≤ 0.60 as low, 0.61–0.80 as moderate, and >0.80 as high [16].

To test the hypothesis that the electronic on-farm milk flow meter (i.e., SLP_MM_) could identify milking liner slips, as verified through the VaDia device (i.e., SLP_V1_ and SLP_V2_), the same approach was used as outlined for trial 1. For the calculation of the test statistics (sensitivity, specificity, PPV, and NPV), SLP_V1_ and SLP_V2_ were considered as the gold standards.

## 3. Results and Discussion

### 3.1. Trial 1—Audible Detection and Milk Flow Meter System

In trial 1, we obtained data from a total of 2039 individual cow milking observations. Among the 2039 cows that contributed one milking observation to the final analyses, 671 (33%) were in their first lactation, 397 (19%) were in their second lactation, and 971 (48%) were in their third or greater lactation. The cows were between 1 and 361 DIM [mean ± standard deviation (SD): 143 ± 109 DIM]. The average values (mean ± SD) of the milking characteristics were as follows: milk yield, 15.9 ± 4.2 kg/milking session; 2-minute milk yield, 7.7 ± 2.4 kg; average milk flow rate, 3.7 ± 0.9 kg/min; and milking unit-on time, 255 ± 56 s. We documented a total of 199 (9.8%) milking liner slips according to audible detection and 126 (6.2%) milking liner slips according to the milk flow meter system. These frequency distributions were comparable to a previous study from our group using the same milk flow meter technology and the same threshold value (12.4%) [17]. Other researchers have used different techniques, such as measuring vacuum fluctuations with vacuum transducers [8,18] or employing audible detection [9]. Spencer and Rogers [18] found that the mean number of liner slips per milking observation was related to the operating vacuum and ranged from 3.9 to 8.8. Baxter et al. [8] studied the relationship between milking machine liner slips and new intramammary infection and reported average numbers of liner slips per milking session of 3.6 and 6.1 for two different milking liners. Rasmussen and Madsen [9] assessed the frequency of audible liner slips and reported that the frequency distribution ranged between 2.4% and 30.0%, depending on the vacuum setting and milking equipment. In this study, a lower operating vacuum was associated with a lower frequency of liner slips [9].

Frequency distributions of milking liner slips according to the three observers (SLP_OB1_, SLP_OB2_, and SLP_OB3_), their overall evaluation (SLP_ANY_), and the on-farm milk flow meter (SLP_MM_) are shown in Table 1. Observer 1 detected the largest number of audible liner slips at the beginning of the milking observation (*n* = 118/2039; 5.8%). We attribute the milking liner slips at the beginning of milking to a bimodal milk flow curve and the associated changes in the short milk tube and mouthpiece chamber vacuums. Due to the inverse relationship between the milk flow rate and the teat-end vacuum [19], the vacuum increases as the milk flow decreases. Bimodality occurs when the contents of the teat cistern are emptied before the alveolar fraction of milk is let down. Additionally, the teat barrel diameter decreases because of lower positive pressure in the teat cistern, resulting in a poor seal between the teat and the milking liner barrel [20]. Consequently, the vacuum from the teat end into the mouthpiece chamber increases. If the forces generated by the increases in the mouthpiece chamber vacuum exceed the friction between the teat and the mouthpiece lip, as well as the inertia of the mouthpiece lip, a liner slip can occur. A smaller proportion of liner slips at the beginning of milking may have occurred due to incorrect attachment or alignment of the milking cluster.

The milk flow meter failed to detect 81 out of the 118 liner slips recorded by observer 1 at the beginning of milking, leading to a false negative ratio of 4.2% (81/1913). We believe that the discrepancy between SLP_OB1_ and SLP_MM_ can be attributed mostly to a relatively short duration of the liner slips, resulting in failure to reach the duration threshold of the milk flow meter system. This idea is supported by the additional 89 and 96 liner slips detected by observer 1 that were not recorded by observers 2 and 3, respectively (Table 1). Observer 2 recorded the second largest number of milking liner slips (*n* = 97, 4.8%), among which 69 (3.4%) were also detected through the milk flow meter. The milk flow meter failed to detect 28 (1.4%) milking liner slips, leading to a false negative ratio of 1.5% (28/1913). Based on our subjective evaluation, liner slips that occurred in the middle of the milking observation were mostly due to cows with asymmetric udder conformation or a nonlactating quarter; these physiological differences led to inadequate milking unit alignment. The audible signs were often lasting throughout the entire observation of observer 2. Observer 3 documented a total of 56 (2.8%) liner slips, 39 of which were also detected by the on-farm milk meter, resulting in the lowest false negative ratio of 0.9% (17/1913). We believe that the milking liner slips recorded at the end of milking can be attributed mostly to overmilking (i.e., the amount of milk extracted from the teat exceeded the milk flow from the gland cistern to the teat cistern [21]) and the same etiology as outlined above for the liner slips associated with bimodality. It should be noted that the milking observation for cows with low milk production was completed before they reached the station of observer 3. This likely increased the false negative rate of 4.4% (87/1983).

The κ values and 95% CIs for the agreement between SLP_MM_ and the audible detection of a machine milking liner slip recorded by the three observers individually (SLP_OB1_, SLP_OB2_, and SLP_OB3_), in combination (SLP_OB1+2_, SLP_OB1+3_, and SLP_OB2+3_), and their overall assessment (SLP_ANY_) are presented in Table 2. Our results indicated fair agreement with SLP_MM_ for SLP_OB1_, moderate agreement for SLP_OB2_, SLP_OB3_, SLP_OB1+2_, SLP_OB1+3_, and SLP_ANY_, and good agreement for SLP_OB2+3_. Using the audible detection by the three observers individually (SLP_OB1_, SLP_OB2_, and SLP_OB3_), in combination (SLP_OB1+2_, SLP_OB1+3_, and SLP_OB2+3_), and their overall assessment (SLP_ANY_) as the gold standards, we evaluated the diagnostic test statistics for using SLP_MM_ to detect a milking liner slip. The diagnostic test statistics indicated low to moderate sensitivity, with values ranging from 0.31 (SLP_OB1_) to 0.71 (SLP_OB2_); high performance for specificity; low to moderate PPV, ranging from 0.29 (SLP_OB1_) to 0.73 (SLP_ANY_); and high NPV, ranging from 0.94 (SLP_ANY_) to 0.99 (SLP_OB2_ and SLP_OB3_). The low to moderate sensitivity is not a surprising result since the detection of liner slips with the milk flow meter was tied to a minimum duration of 30 s, while audible liner slips of any length were recorded. The poor PPV can be attributed to the overall low frequency of liner slips, which gives a higher impact to small deficits in specificity.

### 3.2. Trial 2—Vacuum Recordings and Milk Flow Meter System

In trial 2, we obtained data from a total of 904 individual cow milking observations. Among the 904 cows, 316 (35%) were in their first lactation, 250 (28%) were in their second lactation, and 338 (37%) were in their third or greater lactation. The cows were between 1 and 749 DIM (mean ± standard deviation (SD): 160 ± 105 DIM). The average values (mean ± SD) of the milking characteristics were as follows: milk yield, 14.2 ± 4.0 kg/milking session; 2-minute milk yield, 7.1 ± 2.6 kg; average milk flow rate, 3.5 ± 0.9 kg/min; and milking unit-on time, 237 ± 58 s. We documented a total of 261 (28.9%) milking liner slips according to SLP_V1_, 496 (54.9%) according to SLP_V2_, and 47 (5.2%) according to SLP_MM_ (Table 3). The mean (±SD) value of the average cyclic vacuum fluctuations was 36.8 ± 8.7 kPa. The average number (mean ± SD; range) of events of irregular vacuum fluctuations type 1 and type 2, respectively, was 2.3 ± 10.3 (0–106) and 6.1 ± 18.9 (0–235).

The κ values and 95% CIs for the agreement between SLP_MM_ and SLP_V1_, as well as between SLP_MM_ and SLP_V2_, respectively, were 0.13 (0.07–0.20) and −0.18 (−0.24–0.12), indicating poor agreement. We attribute the observed discrepancies to the differences in the derivation of the milking liner slip data. That is, the VaDia device measures vacuum at a frequency of 200 Hz, whereas the milk flow meter detects air leakages with a minimum duration of 30 s. The diagnostic test statistics for the comparison between SLP_MM_ and SLP_V1_ yielded low sensitivity, 0.17 (0.13–0.22); high specificity, 0.995 (0.986–0.998); high PPV, 0.94 (0.83–0.98); and moderate NPV, 0.75 (0.72–0.78). For the comparison between SLP_MM_ and SLP_V2_, these values revealed a low sensitivity, 0.10 (0.07–0.12); high specificity, 1.0 (0.99–1.0); high PPV, 1.0 (0.92–1.0); and low NPV, 0.48 (0.44–0.51). These findings indicate that the electronic on-farm milk flow meter system under-detected short-duration irregular vacuum fluctuations documented with the VaDia device. The higher frequency of liner slips detected with the VaDia device and the high specificity for this comparison increased the PPV. We conclude that a large proportion of liner slips, including those that may facilitate retrograde movement of milk droplets and bacteria through the teat canal into the mammary gland, are not detected via the milk flow meter system. However, this conclusion remains speculative, as we did not investigate the association of liner slips with mastitis risk.

### 3.3. Study Limitations and Future Research

One limitation of trial 1 was the limited number of observers, which restricted our ability to detect short-duration liner slips, specifically those occurring at the end of the milking observation, resulting in false negative results. Furthermore, some mention should be made of our choice to use multiple combinations of the results obtained from the three observers as the gold standards. We chose this approach to identify possible differences in the sensor-based detection of liner slips throughout a cow milking observation. However, in the absence of current knowledge about the impact of machine milking liner slips on milking performance and mastitis risk, our choice was somewhat arbitrary and did not reflect a true gold standard. However, we believe that SLP_ANY_, which consisted of the sum of assessments from all three observers, most closely resembles the gold standard for audible liner slips in trial 1. Another limitation of trial 1 was that data collection was limited to a single milking session from a single farm. The collection of data from several visits and different farms, as performed in trial 2, could have led to more robust results and increased the generalizability of trial 1.

Machine-milking liner slips have been associated with decreased udder health [5,8]. However, these studies date back several decades, and the results may no longer apply to today’s dairy industry [5,8]. Therefore, studies are needed to investigate the relationship between liner slips and udder health. 

## 4. Conclusions

Our results imply that on-farm milk flow meters reported milking machine liner slips somewhat comparably to audible detection but significantly underreported liner slips compared to vacuum recordings. The on-farm milk flow meter system was best at detecting milking liner slips that occurred in the middle and at the end of milking, but it also underestimated liner slips that occurred at the beginning of milking. To improve the efficacy of automated liner slip detection programs, we recommend lowering the threshold values and the minimum durations needed to detect a liner slip, as well as including a time stamp for each liner slip detection relative to the beginning of milking. However, more research is needed to establish optimum settings for the on-farm milk flow meters. This could help farmers accurately understand the number of liner slips that happen during milking, which can inform their choice of milking liner and vacuum settings. Furthermore, more accurate detection can advance our understanding of the relationship between liner slips and milking performance parameters (e.g., milking unit-on time), as well as the previously reported associations between liner slips and udder health [5,8] in modern dairy operations.

## Figures and Tables

**Table 1:** Number and percentage of milking liner slips detected at 3 observer positions (SLP_OB1_, SLP_OB2_, and SLP_OB3_), an overall evaluation by all 3 observers (SLP_ANY_), and those detected through an electronic on-farm milk flow meter (SLP_MM_) from 2039 individual-cow milking observations at 1 commercial dairy farm. Percentage values may not add up to 100% due to rounding errors.

	SLP_OB1_			SLP_OB2_			SLP_OB3_			SLP_ANY_		
	Present	Absent	Total	Present	Absent	Total	Present	Absent	Total	Present	Absent	Total
SLP_MM_												
Present	37 (1.8%)	89 (4.4%)	126 (6.2%)	69 (3.4%)	57 (2.8%)	126 (6.2%)	39 (1.9%)	87 (4.3%)	126 (6.2%)	92 (4.5%)	34 (1.7%)	126 (6.2%)
Absent	81 (4.0%)	1832 (89.9%)	1913 (93.8%)	28 (1.4%)	1885 (92.5%)	1913 (93.8%)	17 (0.8%)	1896 (93.0%)	1.913 (93.8%)	107 (5.3%)	1806 (88.6%)	1913 (93.8%)
Total	118 (5.8%)	1921 (94.2%)	2039	97 (4.8%)	1942 (95.2%)	2039	56 (2.8%)	1983 (97.3%)	2039	199 (9.8%)	1840 (90.2%)	2039
SLP_OB1_												
Present	-	-	-	29 (1.4%)	89 (4.4%)	118 (5.8%)	22 (1.1%)	96 (4.7%)	118 (5.8%)	118 (5.8%)	0 (0%)	118 (5.8%)
Absent	-	-	-	68 (3.3%)	1853 (90.9%)	1921 (95.2%)	34 (1.7%)	1887 (92.6%)	1921 (94.2%)	81 (4.0%)	1840 (90.2%)	1921 (94.2%)
Total	-	-	-	97 (4.8%)	1942 (95.2%)	2039	56 (2.8%)	1983 (97.3%)	2039	199 (9.8%)	1840 (90.2%)	2039
SLP_OB2_												
Present	-	-	-	-	-	-	39 (1.9%)	58 (2.8%)	97 (4.8%)	97 (4.8%)	0 (0%)	97 (4.8%)
Absent	-	-	-	-	-	-	17 (0.8%)	1925 (94.4%)	1942 (95.2%)	102 (5.0%)	1840 (90.2%)	1942 (95.2%)
Total	-	-	-	-	-	-	56 (2.8%)	1983 (97.3%)	2039	199 (9.8%)	1840 (90.2%)	2039
SLP_OB3_												
Present	-	-	-	-	-	-	-	-	-	56 (2.8%)	0 (0%)	56 (2.8%)
Absent	-	-	-	-	-	-	-	-	-	143 (7.0%)	1840 (90.2%)	1983 (97.3%)
Total	-	-	-	-	-	-	-	-	-	199 (9.8%)	1840 (90.2%)	2039

**Table 2:** Agreement, sensitivity (Se), specificity (Sp), positive predictive value (PPV), and negative predictive value (NPV) between the detection of a milking liner slip with an electronic on-farm milk flow meter and 3 different observers from 2039 individual-cow milking observations at 1 commercial dairy farm. κ values indicate agreement beyond that of chance; corresponding 95% confidence intervals (CIs) are provided for all values. These values were used to predict a milking liner slip through the on-farm milk flow meter as detected by observers at positions 1 (SLP_OB1_), 2 (SLP_OB2_), and 3 (SLP_OB3_), their combined evaluations (SLP_OB1+2_, SLP_OB1+3_, and SLP_OB2+3_), and overall evaluation (SLP_ANY_).

Item	κ (95% CI)	Se	Sp	PPV	NPV
SLP_OB1_	0.25 (0.18–0.34)	0.31 (0.24–0.40)	0.95 (0.94–0.96)	0.29 (0.22–0.38)	0.96 (0.95–0.97)
SLP_OB2_	0.60 (0.52–0.68)	0.71 (0.61–0.79)	0.97 (0.96–0.98)	0.55 (0.46–0.63)	0.99 (0.98–0.99)
SLP_OB3_	0.41 (0.31–0.50)	0.70 (0.57–0.80)	0.96 (0.95–0.96)	0.31 (0.24–0.40)	0.99 (0.99–0.99)
SLP_OB1+2_	0.52 (0.45–0.59)	0.46 (0.39–0.53)	0.98 (0.97–0.98)	0.68 (0.60–0.76)	0.95 (0.94–0.96)
SLP_OB1+3_	0.41 (0.33–0.48)	0.41 (0.33–0.49)	0.97 (0.96–0.97)	0.49 (0.41–0.58)	0.95 (0.94–0.96)
SLP_OB2+3_	0.61 (0.54–0.68)	0.67 (0.58–0.75)	0.97 (0.97–0.98)	0.60 (0.52–0.68)	0.98 (0.97–0.99)
SLP_ANY_	0.53 (0.46–0.60)	0.46 (0.39–0.53)	0.98 (0.97–0.99)	0.73 (0.65–0.80)	0.94 (0.93–0.95)

**Table 3:** Number and percentage of milking liner slips detected through a portable vacuum recording device (SLP_V1_ and SLP_V2_) and those detected using an electronic on-farm milk flow meter (SLP_MM_) among 904 individual-cow milking observations from 2 dairy farms.

	SLP_V1_			SLP_V2_		
	Present	Absent	Total	Present	Absent	Total
SLP_MM_						
Present	44 (4.9%)	3 (0.3%)	47 (5.2%)	47 (5.2%)	0 (0%)	47 (5.2%)
Absent	217 (24.0%)	640 (70.8%)	857 (94.8%)	449 (50.0%)	408 (45.1%)	857 (94.8%)
Total	261 (28.9%)	643 (71.1%)	904	496 (54.9%)	408 (45.1%)	904

## Data Availability

Data are available from the corresponding author upon reasonable request.
